# Supplementary material for: Multi-system dysregulation in placental malaria contributes to adverse perinatal outcomes in mice
Source: Infect Immun. 2025 Jun 5;93(7):e00021-25. doi: 10.1128/iai.00021-25 (PMC12234438; doi:10.1128/iai.00021-25)
Supplement: Fig. S1 — Mosquito bite Plasmodium berghei challenge in non-pregnant and pregnant dams. [file iai.00021-25-s0001.docx]

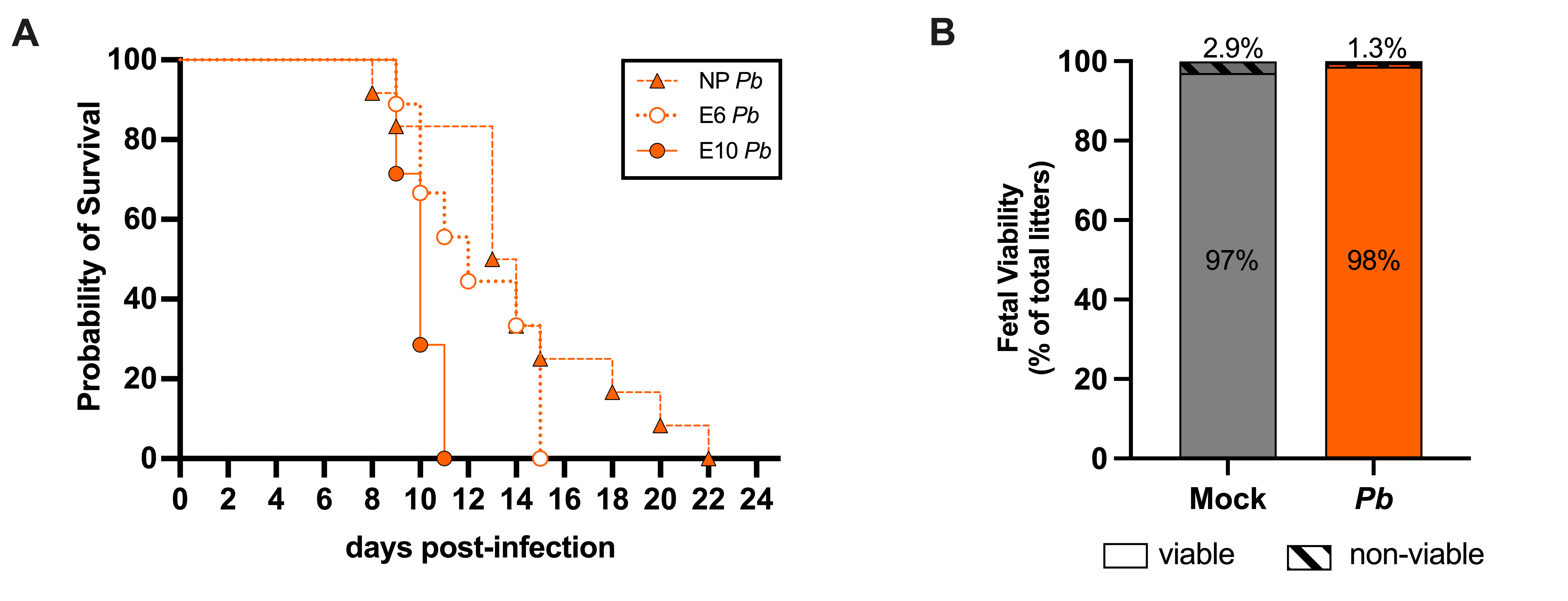


**Supplemental Figure 1. Mosquito bite *Plasmodium berghei* challenge in non-pregnant and pregnant dams. (A)** Kaplan-Meier survival curves for E6 and E10 *Pb*-infected dams. n = 7-12 dams/group across 6 experiments. **(B)** Viability was assessed in fetuses collected at embryonic day 18 by cesarian section in E10 dams. Data are represented as the percentage of viable fetuses out of the total number of fetuses across all litters. Percentages within the solid bar represent viable fetuses while percentages above the striped bar represent non-viable fetuses.
