# Supplementary material for: Multi-system dysregulation in placental malaria contributes to adverse perinatal outcomes in mice
Source: Infect Immun. 2025 Jun 5;93(7):e00021-25. doi: 10.1128/iai.00021-25 (PMC12234438; doi:10.1128/iai.00021-25)
Supplement: Fig. S2 — Fecal microbial taxonomic classification and differential analysis after malaria infection. [file iai.00021-25-s0002.docx]

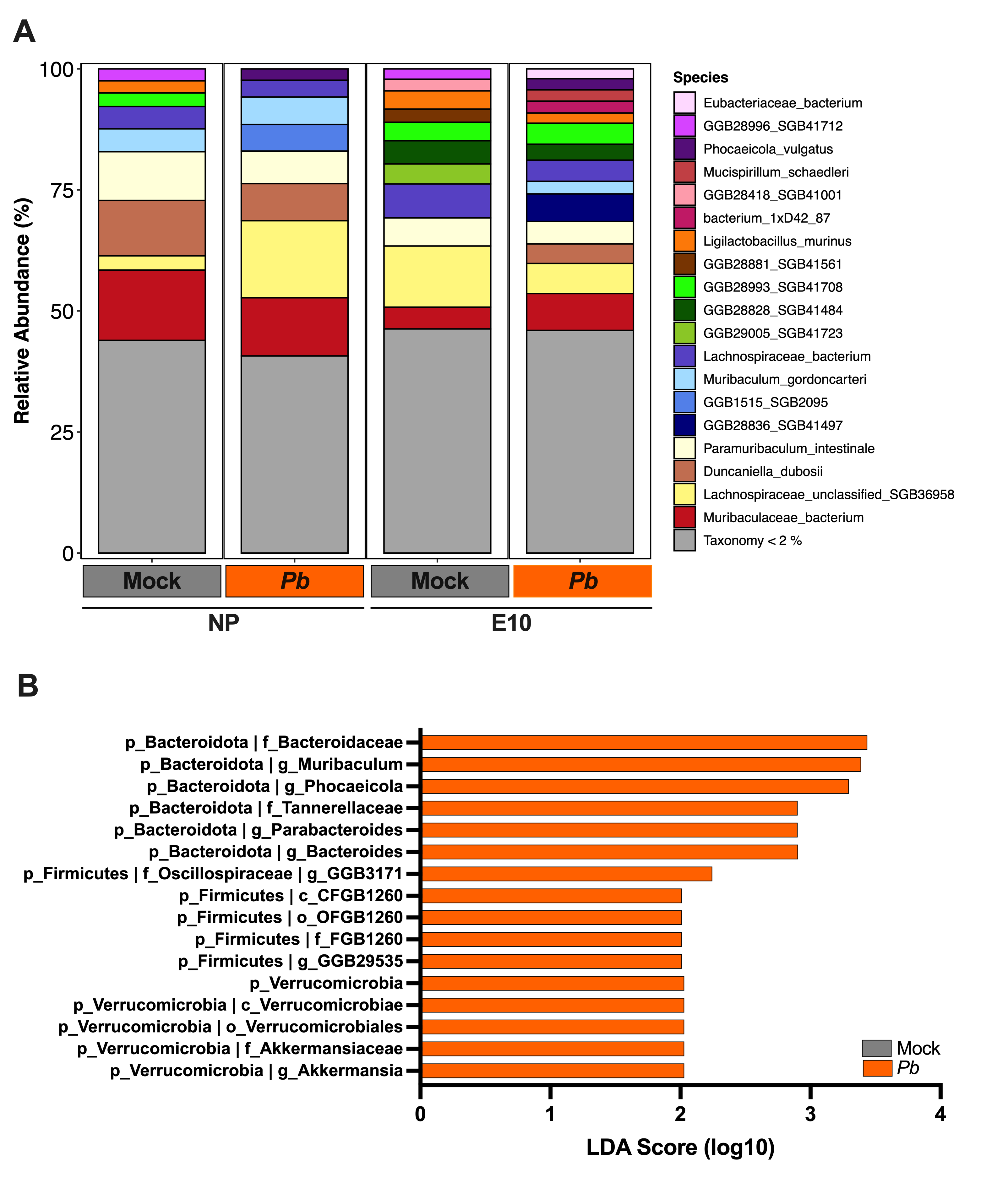
**Supplemental Figure 2. Fecal microbial taxonomic classification and differential analysis after malaria infection. (A)** Relative abundances agglomerated at the species level, separated by infection and pregnancy status. **(B)** Differential analysis comparing mock and *Pb*-infected E10 dams. Linear discriminant analysis score was calculated using linear discriminant analysis effect size (LEfSe). The color represents the infection status (Mock: grey; Pb: orange) for which that species was enriched.
