# Supplementary material for: Multi-system dysregulation in placental malaria contributes to adverse perinatal outcomes in mice
Source: Infect Immun. 2025 Jun 5;93(7):e00021-25. doi: 10.1128/iai.00021-25 (PMC12234438; doi:10.1128/iai.00021-25)
Supplement: Table S1 — Splenic and placental cytokine concentrations for mock- and Plasmodium berghei (Pb)-infected E10 dams at 8 days postinfection. [file iai.00021-25-s0003.docx]

**Supplementary Table 1. Splenic and placental cytokine concentrations for mock- and Plasmodium berghei (Pb)-infected E10 dams at 8 days post-infection.**

|  | **Spleen** | | | **Placenta** | | |
| --- | --- | --- | --- | --- | --- | --- |
| **Cytokine** | **Mock** | **Pb** | **p-value** | **Mock** | **Pb** | **p-value** |
| IFN-α | **33.04 ± 1.82** | **51.46 ± 3.65** | **0.004** | 99.30 ± 16.91 | 95.10 ± 12.33 | 0.840 |
| IFN-γ | **1.19 ± 0.36** | **185.47 ± 66.83** | **0.045** | 0.58 ± 0.16 | 5.40 ± 2.03 | 0.108 |
| IL-28 | 61.3 ± 16.97 | 79.70 ± 13.48 | 0.404 | 30.82 ± 7.30 | 30.09 ± 7.06 | 0.944 |
| IL-1α | **26.79 ±4.16** | **76.61 ± 5.44** | **0.000** | 28.13 ± 9.71 | 22.21 ± 3.46 | 0.537 |
| IL-1β | **6.91 ± 0.62** | **29.62 ± 6.14** | **0.006** | 2.29 ± 0.34 | 1.83 ± 0.13 | 0.215 |
| IL-18 | 4686.21 ± 861.20 | 13208.59 ± 4389.37 | 0.098 | 202.14 ± 24.17 | 282.02 ± 62.61 | 0.302 |
| IL-33 | **6605.82 ± 1576.13** | **1329.58 ± 119.03** | **0.002** | 487.33 ± 120.47 | 333.09 ± 50.59 | 0.220 |
| IL-2 | 5.83 ± 2.16 | 10.37 ± 1.04 | 0.063 | — | — | — |
| IL-2R | **41.95 ± 4.45** | **243.05 ± 33.77** | **0.000** | 5.31 ± 1.28 | 3.81 ± 0.54 | 0.258 |
| IL-4 | **2.25 ± 0.25** | **33.48 ± 4.26** | **0.000** | **0.40 ± 0.05** | **1.40 ± 0.28** | **0.013** |
| IL-7 | 18.58 ± 5.38 | 13.84 ± 2.00 | 0.323 | — | — | — |
| IL-7Rα | **50.39 ± 3.72** | **124.94 ± 16.75** | **0.002** | 18.87 ± 2.42 | 19.70 ± 1.82 | 0.784 |
| IL-9 | 9.17 ± 3.48 | 9.93 ± 1.08 | 0.782 | — | — | — |
| IL-15 | **5.37 ± 0.79** | **371.27 ± 84.85** | **0.002** | 3.13 ± 0.69 | 14.45 ± 4.73 | 0.148 |
| TNF-α | **11.53 ± 1.08** | **41.70 ± 3.24** | **0.000** | 7.51 ± 1.01 | 5.99 ± 0.77 | 0.246 |
| RANKL | 44.55 ± 8.85 | 35.15 ± 5.59 | 0.365 | — | — | — |
| BAFF | 1471.43 ± 255.70 | 1933.54 ± 492.45 | 0.458 | 29.39 ± 3.27 | 65.79 ± 11.40 | 0.144 |
| IL-10 | **10.54 ± 2.38** | **38.59 ± 5.23** | **0.001** | — | — | — |
| IL-19 | **210.29 ± 21.47** | **410.66 ± 44.75** | **0.002** | — | — | — |
| IL-22 | **6.60 ± 0.90** | **38.41 ± 2.48** | **0.000** | 5.49 ± 1.89 | 2.45 ± 0.46 | 0.100 |
| IL-12p70 | **0.57 ± 0.06** | **2.14 ± 0.19** | **0.000** | 0.38 ± 0.05 | 0.39 ± 0.02 | 0.891 |
| IL-23 | 11.15 ± 3.28 | 12.57 ± 1.93 | 0.719 | — | — | — |
| IL-27 | **6.17 ± 0.91** | **22.35 ± 1.27** | **0.000** | 7.83 ± 1.03 | 6.39 ± 0.93 | 0.318 |
| IL-17A | **2.19 ± 0.28** | **41.62 ± 1.87** | **0.000** | 0.98 ± 0.23 | 0.70 ± 0.14 | 0.291 |
| IL-25 | **96.43 ± 16.88** | **299.13 ± 7.94** | **0.000** | 58.83 ± 9.77 | 49.12 ± 6.31 | 0.399 |
| IL-6 | **9.24 ± 1.56** | **53.91 ± 10.69** | **0.003** | 5.71 ± 0.97 | 9.51 ± 1.70 | 0.094 |
| IL-31 | **6.04 ± 1.23** | **56.90 ± 1.64** | **0.000** | 1.70 ± 0.24 | 1.90 ± 0.25 | 0.572 |
| LIF | **7.47 ± 1.36** | **20.84 ± 2.15** | **0.000** | 6.48 ± 0.87 | 4.56 ± 0.81 | 0.131 |
| CCL2 | **27.62 ± 2.03** | **403.15 ± 40.19** | **0.000** | 82.97 ± 17.08 | 102.80 ± 15.77 | 0.411 |
| CCL3 | **9.36 ± 1.84** | **615.98 ± 66.59** | **0.000** | **5.10 ± 0.66** | **3.56 ± 0.28** | **0.036** |
| CCL4 | **11.22 ± 2.72** | **249.77 ± 50.27** | **0.001** | **1.47 ± 0.25** | **2.23 ± 0.23** | **0.041** |
| CCL5 | 983.93 ± 138.21 | 1442.05 ± 160.06 | 0.052 | **12.54 ± 2.60** | **21.37 ± 2.64** | **0.040** |
| CCL7 | **32.53 ± 5.37** | **262.83 ± 28.93** | **0.000** | 57.20 ± 8.00 | 96.43 ± 14.92 | 0.051 |
| CCL11 | 107.34 ± 22.02 | 100.68 ± 21.36 | 0.833 | 49.57 ± 4.96 | 46.30 ± 3.15 | 0.571 |
| CXCL1 | **60.90 ± 15.05** | **131.43 ± 13.95** | **0.004** | **99.19 ± 13.91** | **237.63 ± 26.05** | **0.001** |
| CXCL2 | **5.20 ± 0.42** | **13.56 ± 1.21** | **0.000** | **3.11 ± 0.52** | **6.07 ± 0.97** | **0.027** |
| CXCL5 | **252.66 ± 26.60** | **175.34 ± 10.35** | **0.010** | — | — | — |
| CXCL10 | **35.50 ± 4.41** | **113.33 ± 9.87** | **0.000** | — | — | — |
| BTC | 6.89 ± 1.30 | 8.31 ± 0.75 | 0.335 | — | — | — |
| M-CSF | **6.44 ± 1.52** | **17.88 ± 2.33** | **0.002** | 74.79 ± 32.58 | 24.61 ± 6.14 | 0.109 |
| **Table 3 continued** | | | | | | |
| GM-CSF | **0.92 ± 0.10** | **2.79 ± 0.25** | **0.000** | 0.55 ± 0.11 | 0.63 ± 0.06 | 0.538 |
| G-CSF | 3.80 ± 0.65 | 6.68 ± 1.12 | 0.058 | 10.17 ± 3.06 | 7.05 ± 2.51 | 0.440 |
| IL-3 | **0.29 ± 0.06** | **1.22 ± 0.17** | **0.000** | 0.18 ± 0.04 | 0.26 ± 0.07 | 0.353 |
| VEGF-A | 19.10 ± 0.94 | 21.72 ± 1.78 | 0.253 | 16.28 ± 1.40 | 15.36 ± 0.78 | 0.551 |
| IL-13 | **7.13 ± 1.09** | **34.80 ± 3.51** | **0.000** | — | — | — |
| IL-5 | **15.78 ± 1.04** | **30.39 ± 2.03** | **0.000** | 1.83 ± 0.43 | 1.98 ± 0.39 | 0.798 |
| E10 dams euthanized at 8 dpi. Data are mean ± SEM, n = 7-9 dams/group, from 2 replicates. Bold indicates a statistically significant difference between mock and Plasmodium berghei groups, based on unpaired t-test, p-value <0.05. Dashes (—) indicates analytes which were below the limit of detection. | | | | | | |
