# Supplementary material for: Multi-system dysregulation in placental malaria contributes to adverse perinatal outcomes in mice
Source: Infect Immun. 2025 Jun 5;93(7):e00021-25. doi: 10.1128/iai.00021-25 (PMC12234438; doi:10.1128/iai.00021-25)
Supplement: Table S2 — Correlation between bacterial species and pregnancy outcomes in mock- and Plasmodium berghei (Pb)-infected E10 dams at 8 days postinfection. [file iai.00021-25-s0004.docx]

**Supplementary Table 2. Correlation between bacterial species and pregnancy outcomes in mock- and Plasmodium berghei (Pb)-infected E10 dams at 8 days post-infection.**

| **Group** | **Measure** | **Taxonomy (Spearman Coefficient, p value)** |
| --- | --- | --- |
| Offspring Outcomes | Viable Pups | bacterium 1xD42 87 (0.36, *p=0.037*) |
|  | Pup Mass | Lachnospiraceae bacterium (0.54, *p=0.022*) |
|  | Placental Mass | Lachnospiraceae unclassified SGB36958 (0.44, *p=0.048*) |
|  | Placental Efficiency | Lachnospiraceae bacterium (0.69, *p=0.009*) |
| Placenta Cytokines | IL-4 | *Mucispirillum schaedleri* (0.83, *p=0.007*) |
| Placenta Chemokines | CCL4 | bacterium 1xD42 87 (0.73, *p=0.005*) |
|  | CCL5 | *Clostridia* bacterium (0.58, *p=0.046*) |
|  | CXCL2 | Oscillospiraceae bacterium (0.54, *p=0.042*)  *Acetatifactor muris* (0.70, *p=0.015*) |
| Splenic IFN | IFN alpha | *Clostridia* bacterium (-0.83, *p=0.013*)  *Anaerotruncus sp* 1XD42 93 (0.79, *p=0.034*) |
|  | IFN gamma | *Candidatus Arthromitus sp SFB* mouse (0.45, *p=0.050*)  *Parasutterella excrementihominis* (0.53, *p=0.038*) |
|  | IL-28 | *Bacteroides xylanisolvens* (-0.60, *p=0.042*)  *Bacteroides thetaiotaomicron* (-0.65, *p=0.046*) |
| Splenic IL-1 | IL-18 | Eubacteriaceae bacterium (-0.10, *p=0.009*) |
| Splenic Common 𝜸 chain | IL-2 | *Candidatus Arthromitus sp SFB* mouse (0.83, *p=0.006*)  *Adlercreutzia caecimuris* (0.77, *p=0.034*)  *Parasutterella excrementihominis* (0.50, *p=0.037*) |
|  | IL-7R alpha | Clostridia bacterium (-0.67, *p=0.041*) |
|  | IL-9 | bacterium 1xD42 87 (-0.85, *p=0.032*)  *Bacteroides xylanisolvens* (-0.73, *p=0.041*)  *Bacteroides thetaiotaomicron* (-0.82, *p=0.041*) |
| Splenic TNF | RANKL | *Parasutterella excrementihominis* (0.61, *p=0.019*) |
|  | BAFF | *Parasutterella excrementihominis* (0.79, *p=0.009*) |
| Splenic IL-10 | IL-10 | *Parasutterella excrementihominis* (0.58, *p=0.013*) |
|  | IL-19 | bacterium 1xD8 6 (0.62, *p=0.040*) |
| Splenic IL-12 | IL-23 | *Candidatus Arthromitus sp SFB* mouse (0.73, *p=0.046*)  *Adlercreutzia caecimuris* (0.75, *p=0.008*) |
| Splenic IL-6 | IL-6 | *Clostridiaceae bacterium* (0.47, *p=0.026*) |
| Splenic Chemokines | CCL4 | Clostridia bacterium (-0.68, *p=0.019*) |
|  | CCL5 | bacterium 1xD42 87 (0.75, *p=0.021*) |
|  | CCL11 | Lachnospiraceae bacterium A2 (0.75, *p=0.007*) |
|  | CXCL2 | bacterium 1xD8 6 (0.53, *p=0.031*) |
|  | CXCL10 | Clostridia bacterium (-0.65, *p=0.048*) |
| Splenic Growth Factors | Betacellulin | *Parabacteroides distasonis* (-0.77, *p=0.045*)  *Bacteroides xylanisolvens* (-0.77, *p=0.010*)  *Bacteroides thetaiotaomicron* (-0.82, *p=0.008*) |
|  | G-CSF | Muribaculaceae bacterium (-0.65, *p=0.027*)  *Duncaniella dubosii* (-0.64, *p=0.041*)  Clostridiaceae bacterium (0.62, *p=0.021*)  *Adlercreutzia caecimuris* (0.73, *p=0.038*) |
|  | M-CSF | Clostridia bacterium (-0.65, *p=0.044*) |
|  | VEGF-A | Eubacteriaceae bacterium (0.24, *p=0.027*)  Clostridia bacterium (-0.70, *p=0.016*)  *Adlercreutzia caecimuris* (0.84, *p=0.019*) |
| Splenic Misc | IL-13 | Clostridia bacterium (-0.73, *p=0.038*) |

| Placental efficiency was calculated by dividing the mean pup mass by the mean placental mass. |
| --- |
